# Development of ventricular fibrillation after implantation of a biventricular implantable cardioverter defibrillator: what is the mechanism?

**DOI:** 10.1002/ccr3.1020

**Published:** 2017-06-02

**Authors:** Diego Chemello, Fernando Pivatto Júnior, Maurício Pimentel, Leandro Zimerman

**Affiliations:** ^1^ Hospital de Clínicas de Porto Alegre (HCPA)Porto AlegreBrazil; ^2^ Universidade Federal do Rio Grande do Sul (UFRGS)Porto AlegreBrazil

**Keywords:** Defibrillators, heart failure, implantable, syncope, ventricular fibrillation

## Abstract

Syncopal spells in heart failure patient with cardiovascular implantable electronic devices (CIED) require multiple assessments. T‐wave oversensing is a well‐described phenomenon that remains significant in modern implantable cardioverter defibrillators (ICD) systems. It can lead to inappropriate therapies and loss of biventricular pacing in those with cardiac resynchronization devices. Strategies to overcome this problem are important.

## Case Report

A 51‐year‐old man with a recently implanted biventricular implantable cardioverter defibrillator (CRT‐D) (Lumax 340 HF‐T; Biotronik Inc., Berlin, Germany) at the year of 2013 presented to the outpatient clinic reporting an episode of syncope that occurred during regular daily activities. The patient had nonischemic dilated cardiomyopathy (DCM), left ventricular ejection fraction (LVEF) of 23%, left bundle branch block (LBBB) with a QRS duration of 160 msec, and New York Heart Association (NYHA) class III symptoms despite optimal medical treatment. He had no history of syncope or ventricular arrhythmias.

Device implantation was uneventful and both right ventricular (RV) and left ventricular (LV) leads were successfully positioned, respectively, in the apex toward the interventricular (IV) septum and in a posterolateral vein in the coronary sinus. T‐wave oversensing was not observed at the implantation (true bipolar RV lead sensing). Defibrillation threshold (DFT) testing was not performed. The R‐wave amplitude was 8.2 mV in the RV lead. The pacing parameters were programed as follows: DDD mode with biventricular pacing at 60–130 beats/min, bipolar LV pacing 5 msec ahead of RV, dynamic atrioventricular (AV) interval (120–150 msec), and post‐ventricular atrial refractory period (PVARP) of 250 msec. RV sensitivity was programed to the standard mode with a minimum threshold of 0.8 mV. Tachycardia detection was programed in two zones: a ventricular tachycardia (VT) monitor zone at 167–213 beats/min and a ventricular fibrillation (VF) zone at 214 beats/min (detection counter, 15 out of 18 beats) with burst pacing before charging, followed by 40‐Joule (J) shocks (40 J, 40 J, and 6 × 40 J).

**Figure 1:**
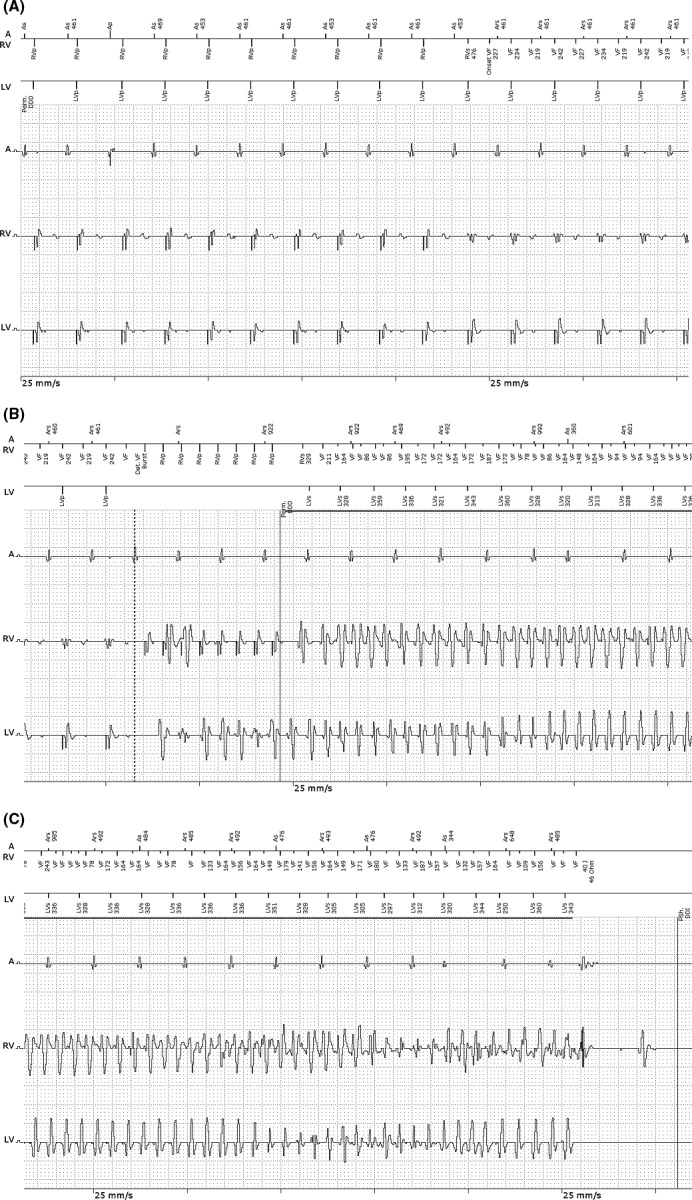
Electrogram (EGM) obtained from the biventricular implantable cardioverter defibrillator upon interrogation. Panels A, B, and C are continuous. The top EGM channel (A) is the atrial channel, middle line (RV) is the right ventricular channel, and bottom line (LV) is the left ventricular channel. Marker channels and chamber intervals are shown on top of the EGMs with atrial markers above the first line. (A) shows the beginning of VF detection, represented by *Onset VF* in the marker channels. (B) shows the time when the VF detection criteria were met (*Det. VF* in the marker channel, corresponding to the dashed vertical line), followed by the delivery of burst pacing (*Burst* in the marker channel). The solid horizontal line represents device charging in the presence of continuing tachyarrhythmia in the VF zone. (C) shows the time when a 40‐J shock successfully terminated the tachycardia. A, right atrium; RV, right ventricle; LV, left ventricle; Ars, atrial refractory period; As, atrial sensing; VF, ventricular fibrillation; RVp, right ventricular pacing; LVp, left ventricular pacing; LVs, left ventricular sensing.

Upon CRT‐D interrogation, one episode of ventricular arrhythmia was retrieved from the device memory. This coincided with the reported episode of syncope (Fig. 1). The episode was classified into the VF zone (Fig. 1A). After the detection criteria were met, burst pacing was performed before charging (Fig. 1B). Since the device was still detecting VF after burst pacing (ATP, 88% of tachycardia cycle length), a single 40‐J shock was delivered and VF was terminated (Fig. 1C). What is the diagnosis of such an event? What strategies should be implemented to avoid further episodes?

## Discussion

**Figure 2:**
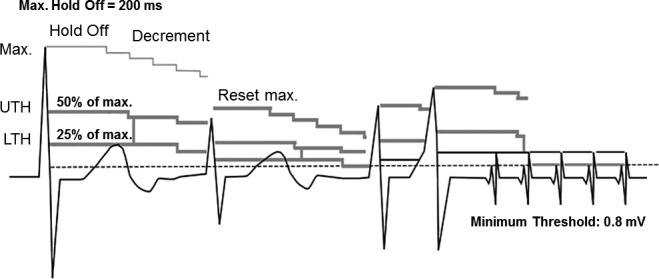
Representation of right ventricular (RV) automatic sensitivity control with the standard mode of Biotronik Lumax 340 HF‐T devices. UTH, upper threshold; LTH, lower threshold. Adapted from Lumax 300/340 Technical Manual.

The analysis of intracardiac electrograms (EGMs) obtained at the beginning of the episode (Fig. 1A) suggests that the initial rhythm was probably sinus tachycardia, based on gradual acceleration with PP intervals driving the VV intervals and biventricular pacing. When the atrial cycle length rose above the programed upper rate (460 msec = 130 bpm), there was loss of AV synchrony (atrial sensing triggering ventricular pacing), manifested as an increase in the AV interval. Since AV conduction was normal, AV lengthening promoted intrinsic ventricular conduction and, consequently, an RV sensed event. The next event observed in the RV EGM occurred at 227 msec and had a different morphology. According to the programed settings, a tachyarrhythmia was detected in the VF zone (*Onset VF*). The subsequent events observed in the RV EGM continued to reproduce this alternating electrocardiographic morphology, a finding compatible with T‐wave oversensing (TWO). Looking more carefully, the T‐waves could be seen on the RV EGM channel even before the *Onset VF* marker came into sight. However, they were not counted because the sensing blanking period after an RV paced event was programed to 300 msec (standard setting of Biotronik devices). Differently, after an RV sensed event, the nominally programed *standard* sensitivity setting to determine the upper threshold (UTH) is calculated as 50% of the R‐wave amplitude and the lower threshold (LTH) as 25% of the R‐wave amplitude, based on a sensed R‐wave. After a sensed event, the UTH remains on hold for 360 msec. Then, the sensed signal changes to the LTH with enhanced sensitivity so as to sense a subsequent low‐amplitude VF event. In the beat‐to‐beat adjustment, each sensed amplitude is measured again and both the UTH and the LTH are reset accordingly. A minimum threshold can be set to protect the input filter from excessive sensitivity (Fig. 2).

**Figure 3:**
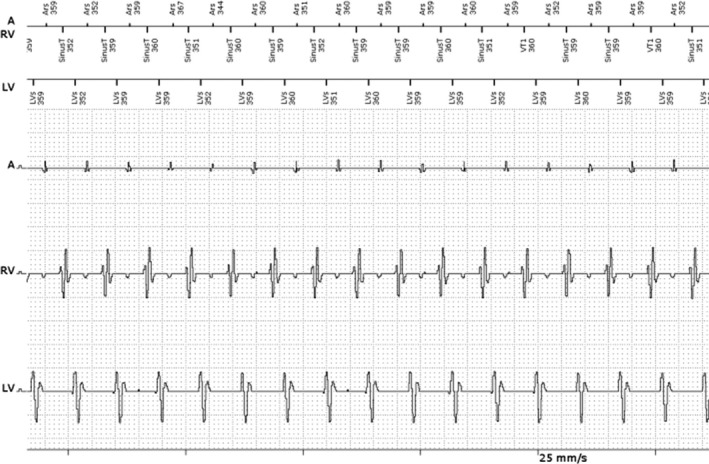
Electrogram (EGM) obtained from the biventricular implantable cardioverter defibrillator upon interrogation during exercise. The top EGM channel (A) is the atrial channel, middle line (RV) is the right ventricular channel, and bottom line (LV) is the left ventricular channel. Marker channels and chamber intervals are shown on top of the EGMs with atrial markers above the first line. There is sinus tachycardia without biventricular pacing. A, right atrium; RV, right ventricle; LV, left ventricle; Ars, atrial refractory period; SinusT, sinus tachycardia; LVs, left ventricular sensing.

Because of the programed upper LV tracking rate of 460 msec, the triggering of RV sensed beats was maintained on an alternating basis (every other RV sensed event). After meeting the VF detection criteria, burst pacing was performed before charging (*Det. VF Burst* marker) (Fig. 1B). However, this burst pacing was performed during sinus tachycardia and therefore induced VF (second pacing spike during burst pacing – T‐wave pacing). After charging the device and confirming tachycardia (a single beat in the VT1 zone up to 2 sec after device charging), a 40‐J shock successfully terminated VF (Fig. 1C). Other EGMs observed during regular activities also confirmed sinus tachycardia and showed loss of biventricular pacing (Fig. 3).

The present case illustrates inappropriate ventricular tachyarrhythmia detection due to TWO, which is a well‐known phenomenon. However, the case also highlights a particularly unusual episode of inappropriate burst pacing leading to VF in a stable patient. In this case, TWO was a consequence of changes in ventricular repolarization in the absence of RV pacing. The arrhythmogenic effects of inappropriate burst pacing during sinus tachycardia caused a true VF episode and syncope. Fortunately, the delivered shock restored sinus rhythm. There were no evidence of drug use or electrolyte imbalance.

Misleading tachycardia detection and inappropriate shocks can occur in up to 21% of patients with an implantable cardioverter defibrillator (ICD) or CRT‐D although more recent trials have reported only 5.9% of inappropriate shocks in patients with nonischemic cardiomyopathy 1. Most of these inappropriate therapies are secondary to atrial fibrillation/flutter, sinus tachycardia, and supraventricular tachycardia. The overall incidence of ventricular oversensing ranges from 4% to 25% in unselected ICD patients. Recent studies have shown that ventricular oversensing accounts for 8.5% of inappropriate shocks and tends to occur soon after device implantation 2. A number of mechanisms may be responsible for TWO, including electrolyte abnormalities, drug use, and changes in sympathetic tone, among others 3. Also, TWO occurs more often after paced beats than spontaneous beats, and this has been attributed to polarization voltage 4. Seegers et al. showed significant device‐related differences in the incidence of ventricular oversensing between manufacturers, with the highest incidence associated with Biotronik devices connected to integrated bipolar leads 5. Maesato et al. also showed that T‐wave amplitudes in ICD recipients with Brugada syndrome were higher than those in non‐Brugada patients, regardless of high‐pass filter settings 6.

It is important to emphasize that the CRT‐D programing was state of the art at the time of the implantation, but it is not in accordance with the latest expert consensus (higher rates for tachycardia detection and increased detection time), which could potentially avoid the inappropriate shock mentioned. Additionally, the programed upper rate was quite low for a young patient. These factors address the importance of a program individualization for each patient 7.

Although TWO leading to inappropriate ICD therapy has been previously reported, this is the first report to show both TWO and device‐induced VF after inappropriate burst pacing. To avoid further episodes of TWO and inappropriate therapies, the device was reprogramed as follows:

- Right ventricular sensitivity was reprogramed from *Standard* to *Enhanced T‐wave Suppression*. This mode was developed to optimize the sensing function, preventing TWO in three ways. First, by increasing the UTH from 50% to 75% of the measured R‐wave while maintaining the LTH at 25%. Second, by increasing high‐pass filtering from 10 to 20 Hertz. Finally, by not recalculating the UTH and LTH if the 100% line is not exceeded.
- DDD upper tracking rate was increased from 130 to 160 beats/min to allow RV pacing during sinus tachycardia.

The potential risks of true VF undersensing were considered. A DFT testing was offered and deferred by the patient. The dose of metoprolol succinate was increased up to the maximum tolerated (i.e., 75 mg/day). An exercise test was performed without any evidence of TWO.

After a 3‐year observation period following CRT‐D reprograming, the patient remains asymptomatic. Device follow‐up has shown no episodes of TWO or inappropriate therapies.

## Authorship

DC and FPJ: Writing of the manuscript. DC, FPJ, MP, and LZ: Critical revision of the manuscript.

## Conflict of Interest

None declared.
